# The Impact of the 2002 Delaware Smoking Ordinance on Heart Attack and Asthma

**DOI:** 10.3390/ijerph7124169

**Published:** 2010-12-02

**Authors:** John Moraros, Yelena Bird, Shande Chen, Robert Buckingham, Richard S. Meltzer, Surasri Prapasiri, Luis H. Solis

**Affiliations:** 1 School of Public Health, University of Saskatchewan, 107 Wiggins Road, Saskatoon, Saskatchewan, S7N 5E5, Canada; E-Mails: yelena.bird@usask.ca (Y.B.); b.buckingham@usask.ca (R.B.); meltzer@zianet.com (R.S.M.); surasri@nmsu.edu (S.P.); luisolis@hotmail.com (L.H.S.); 2 Department of Biostatistics, University of North Texas Health Science Center at Fort Worth, 3500 Camp Bowie Blvd., Fort Worth, TX 76107, USA; E-Mail: chande.chen@unthsc.edu

**Keywords:** acute myocardial infarction, asthma, smoking, secondhand smoke exposure, prevention, health promotion

## Abstract

In the United States, smoking is the leading cause of death - having a mortality rate of approximately 435,000 people in 2000—accounting for 8.1% of all US deaths recorded that year. Consequently, we analyzed the Delaware Hospital Discharge Database, and identified state and non-state residents discharged with AMI or asthma for the years 1999 to 2004. Statistical data analysis compared the incidence of AMI or asthma for each group before (1999–2002) and after (2003–2004) the amendment. As a result, we found that pre-ordinance and post-ordinance quarterly rates of AMI for Delaware residents were 451 (se = 21) and 430 (se = 21) respectively, representing a 4.7% reduction. Over the same time period, there was negligible change in the incidence of AMI for non-Delaware residents. After adjusting for population growth, the Risk Ratio (RR) for asthma in Delaware residents post-ordinance was 0.95 (95% CI, 0.90 to 0.999), which represented a significant reduction (P = 0.046). By comparison, non-Delaware residents had an increased RR for asthma post-ordinance of 1.62 (95% CI, 1.46 to 1.86; P < 0.0001).The results suggest that Delaware’s comprehensive non-smoking ordinance effectively was associated with a statistically significant decrease in the incidence of AMI and asthma in Delaware residents when compared to non-Delaware residents.

## 1. Introduction

In the United States (US), smoking is the leading cause of death - having a mortality rate of approximately 435,000 people in 2000—accounting for 8.1% of all US deaths recorded that year [1]. Consequently, the detrimental effects of smoking and exposure to secondhand smoke (SHS) have been well established in medical literature, which demonstrated a strong association with cardiovascular disease and acute myocardial infarction (AMI) [2]. As a result, smoking and SHS have been shown to be dangerous to respiratory health of all people with special consideration given to those afflicted with respiratory disease—especially asthma sufferers [3,4]. Decades of evidence regarding the hazards of smoking and exposure to SHS have led many communities across the western world to institute comprehensive non-smoking ordinances (which include the workplace, restaurants, and bars) and has led others to amend partial ordinances to become comprehensive.

Recently, four different studies have demonstrated significant decreases in the incidence of AMI after the enactment and implementation of a comprehensive local smoke-free ordinance [5–7]. The cities of Pueblo, Colorado, and Helena, Montana in the US have shown between 27–40% decreases in the incidence of AMI. Further, the Piedmont region of Italy has found an 11% decrease in AMI in individuals under the age of 60 after the implementation of a strong anti-smoking ban. In addition, a recent study conducted in the metropolitan area of Toronto, Canada, concluded that not only incidences of AMI were reduced, but also asthma, stroke, pneumonia, and chronic obstructive pulmonary disease were also affected [8]. Interestingly, all four studies reported a significant improvement in cardiovascular health benefits in locations which had not initiated comprehensive smoking ordinances previously. In addition, studies that include asthma, such as Rayens *et al.*, have shown a 22% decrease in asthma emergency department visits post-law [9]. Therefore, these findings confirmed the long-standing assumption that comprehensive smoking ordinances may represent a practical and effective tool in the fight to reduce the health care burden due to cardiovascular diseases.

Currently, eight US states have implemented comprehensive non-smoking ordinances [10]. Notably, 54.8% of the US population is protected by at least a partial non-smoking ordinance (workplaces and/or restaurants and/or bars), including 21.7% who are protected by a comprehensive ordinance (covering workplaces, restaurants, and bars) [11]. However, previous studies did not discuss the potential health benefits derived from the legislative change from a partial to a comprehensive ban as well as the consequences of statewide non-smoking ordinances. As a result, the purpose of this study was to examine and determine the effects of a comprehensive non-smoking ban on the hospitalization rates of patients due to AMI and asthma among residents and non-residents of the state of Delaware, in the US.

## 2. Experimental Section

### 2.1. Setting

According to US census data, the population of Delaware was 783,600 in 2000 and estimated to be 843,524 in 2005 [12–14]. Additionally, over the time period from 1999 to 2004, Delaware had a higher percentage of smokers when compared to the national US average (25.4% versus 22.8% in 1999 and 24.4% versus 20.9% in 2004) [15,16].

### 2.2. Ordinance

The *Delaware Clean Indoor Air Act* of 1994 became comprehensive in 2002. It was amended to encompass all enclosed indoor areas accessible to the general public, which included restaurants, bars, and casinos [17]. Prior to the amendment, such hospitality venues were exempt from the smoking ban. At the time, the new ordinance was considered one of the most aggressive non-smoking bans in the country. Accordingly, the Delaware Department of Public Health reported 99.6% compliance in bars and restaurants, and the Delaware Department of Labor reported 100% compliance in other workplaces in the first year of the new regulation [18].

### 2.3. Delaware Database

The state of Delaware hospital discharge database is maintained by the Delaware Department of Health and Social Services. Accordingly, all non-federal, acute-care, and short stay Delaware hospitals report their inpatient discharge information to the Department of Health. Those hospitals or hospital systems include A.I duPont, St. Francis, Christiana Care Health System (Wilmington and Christiana Care), Bayhealth Medical System (Kent General and Milford), Beebe and Nanticoke [18]. Furthermore, there is only one federal hospital in Delaware which is not included in the database – the Wilmington VA Medical Center.

### 2.4. Data Collection

The authors contacted the Delaware Department of Health and Social Services and a formal request was made to the Delaware hospital discharge database for the years 1999 to 2004 (the most current data available). We requested and received the primary discharge diagnosis of AMI and asthma patients based on the international classification of the disease (ICD-9-CM code: 410 and 493) for both Delaware and non-Delaware resident patients over the age of 18. Other information requested was: age, sex, quarter of discharge, and state of residence. Patient anonymity and confidentiality was preserved since unique identifiable patient information was neither requested nor provided to the investigators. We included this in the primary analysis. Accordingly, the data was provided in an Excel spreadsheet format. From this data, we extracted the quarterly numbers pertaining to AMI and asthma based on the ICD-9 diagnosis codes. Moreover, we excluded data for 4th quarter of 2002 because the smoking ordinance took effect in November of that year.

### 2.5. Statistical Analysis

The statistical analysis included both AMI and asthma rates for both Delaware and non-Delaware residents discharged from state hospitals in the database. The population of Delaware had undergone some change from 1999 to 2004 (census data estimated a 7.6% increase from 2000 to 2005). We used population data from Delaware Vital Statistics Report from 2000 to 2004, and estimated the 1999 population [19]. From the 2004 report, the proportion of females in Delaware in 2000 was 51.4% and the projected proportion of females in 2010 was 51.5%. Therefore, the effect of sex can be ignored. The vital reports do not have direct information regarding the mean age in the state. Thus, we looked over the proportion for ages 65 and older. This proportion slightly increased from 13.0% in 2000 to 13.8% in 2004. Due to a relatively short time interval (24 quarters), we did not adjust for the proportion of elderly, which seems to be slightly conservative. The primary outcome variables examined were AMI and asthma quarterly counts. As a result, primary analyses for Delaware residents were based on the Poisson regression model with adjustment for population. However, the primary analyses for non-Delaware residents could be only based on the Poisson regression model without adjustment for population. Obviously, the non-Delaware data were from a much smaller ‘population’. Therefore, when comparing results from Delaware and non-Delaware residents, we are not only interested in whether it is statistically significant but also pay attention to the magnitude of the relative risk (RR).

First, Poisson regression models were fitted for the number of AMI and asthma events by taking into consideration both the ordinance and seasonal effects. If the seasonal effect was not significant, a simpler Poisson regression model - with only the ordinance effect—was used. Accordingly, this was equivalent to comparing Poisson distributions before and after the ordinance. Resultantly, the risk ratio (RR) for the ordinance (before versus after) was reported together with a 95% confidence interval. If seasonal effect was not included, quarterly rates before and after the smoking ordinance were estimated together with the standard error.

Analyses was performed separately for AMI and asthma cases for both Delaware and non-Delaware residents. Additionally, for AMI, the pre-ordinance and post-ordinance quarterly rates were subtracted to determine the average quarterly decrease. This number was then multiplied by the number of post-ordinance quarters (*n =* 8) to determine the two-year post-ordinance decrease in AMI.

Finally, we included a linear trend term in the Poisson regression models for our secondary analyses. We also used the data prior the ordinance to check for a temporal trend. These analyses are exploratory and have certain limitations. Since the population is growing in general, the Poisson regression without adjustment for the population for non- Delaware residents seems to be conservative with respect to the ordinance effect.

## 3. Results

### 3.1. AMI

After the amendment of the *Delaware Clean Indoor Air Act* at the end of 2002, a statistically significant reduction in the incidence of AMI for Delaware residents was observed (p < 0.001) based on a Poisson regression model adjusted by season (with adjustment for the population). The RR for the post-ordinance vs. the pre-ordinance AMI was found to be 0.91 (95% CI, 0.87 to 0.95). Seasonal effect was not found to be significant (p = 0.154) for AMI and therefore, a Poisson regression model was fitted with only the ordinance effect, which gave similar results—RR for the post-ordinance *vs.* the pre-ordinance being 0.91 (p < 0.001, 95% CI, 0.88 to 0.95) (Tables 1 and 2).

Before the smoking ordinance was enacted in Delaware, quarterly rates of AMI were 451 (se = 21) and subsequently decreased to 430, (se = 21) demonstrating a 4.7% reduction. Taking into account the difference between pre-ordinance and post-ordinance quarterly rates of AMI, we observed a noticeable decline in AMI rates after the enforcement of the statewide comprehensive ordinance. Consequently, this resulted in approximately 169 fewer AMI cases over the 8 quarter post-ordinance period.

In comparison, similar calculations performed for non-Delaware residents included in the Delaware Discharge Database showed no ordinance effect (p = 0.726) based on population adjusted model with season effect. Consequently, non-Delaware residents discharged from Delaware hospitals had similar pre-ordinance and post-ordinance AMI risk (RR 0.98, 95% CI, 0.90 to 1.08, *P* = 0.726) (Tables 1 and 2).

To examine possible temporal trend, we first fitted a Poisson regression model including season and a linear trend term (with adjustment for population) based on the prior ordinance Delaware resident data. The linear trend term turned out to be insignificant (p = 0.924). Again, a model including ordinance, season and linear trend using pre and post ordinance data showed that the linear trend is not significant (p = 0.557).

Therefore, for Delaware residents there was a statistically significant decrease in the number of AMI discharges post-ordinance. However, for non-Delaware residents ordinance did not show a significant impact on AMI.

### 3.2. Asthma

When examining asthma rates in Delaware residents (adjusted for population) the results show a significant ordinance effect (p = 0.046) together with a strong seasonal effect (p < 0.001). The RR for ordinance (post *vs.* pre) was 0.95 with a 95% CI, 0.90 to 0.99, which represents a significant reduction in quarterly asthma discharges from pre-ordinance to post-ordinance (Table 2 and 3).

In comparison, for asthma in non-Delaware residents, both the seasonal ((p < 0.001) and ordinance effect (p < 0.001) were significant. The RR (post *vs.* pre) for ordinance is estimated at 1.62 (95% CI, 1.41 to 1.86), indicating a significant increase in the rates of asthma from pre-ordinance to post-ordinance (Table 2 and 3).

Similar to AMI, a Poisson regression model including season and a linear trend term (with adjustment for population) based on the prior ordinance Delaware resident data was fitted. The linear trend term is significant (p < 0.001) and so is the season (p < 0.001). The RR (a year *vs.* previous year) was 0.95 (95% CI 0.93 to 0.98); indicating a decreasing asthma rate in time. In a model including ordinance, season and linear trend (using pre and post ordinance data), ordinance (p = 0.025), season (p < 0.001) and linear trend (p < 0.001) are all significant. The RR for linear trend (year) is 0.95 (95% CI 0.92 to 0.97). However, ordinance showed a revise effect (post *vs.* pre RR = 1.12, 95% CI 1.01 to 1.24). Similar analyses were performed for non-Delaware resident data. For pre ordinance data, the linear trend is not significant (p = 0.064). For both pre and post ordinance data, the season (p < 0.001) and linear trend (year) (p = 0.036) are significant but the ordinance is not significant (RR = 1.23, p = 0.161). However, the temporal trend for asthma was increasing in time (RR for year = 1.09, 95% CI 1.01 to 1.19).

Combining the results from the analyses with and without adjustment of temporal trend, the RR for ordinance for Delaware residents ranged from 0.95 (without temporal trend) to 1.12 (with temporal trend), while the one for non-Delaware residents ranged from 1.23 (with temporal trend) to 1.62 (without temporal trend). It is also noted that the temporal trends obtained for Delaware and non-Delaware data are in opposite directions (decreasing for Delaware and increasing for non-Delaware). As a result, the Delaware residents are likely to be protected for asthma when compared with non-Delaware residents, during the post-ordinance period.

## 4. Discussion

Currently, eight states have comprehensive statewide non-smoking ordinances in the US [9]. Additionally, over half of the entire US population is covered by at least a partial non-smoking ordinance [10]. Accordingly, previous studies have addressed some of the health benefits of smoking bans, which found significant decreases in the incidence of AMI when comprehensive non-smoking ordinances were enacted in the absence of prior partial smoking bans (Helena, Pueblo, and Piedmont) [5–7]. However, these studies did not address the issues of *statewide* non-smoking ordinances or *partial* to *comprehensive* smoking bans. Consequently, the present study addressed both issues and found statistically significant decreases in both the AMI and asthma discharge rates for a statewide non-smoking ordinance that was amended from a partial to a comprehensive ban on smoking. In addition, the asthma results of the present study are shown to have an added health benefit of non-smoking ordinances that had not been previously analyzed.

### 4.1. AMI

With the amendment of the *Delaware Clean Indoor Air Act* in 2002, the relative risk of having an AMI after adjusting for population growth was 9% less when compared to the pre-ordinance risk. It is estimated that as a direct consequence of the ordinance, it can be calculated that there have been approximately 340 fewer AMI discharges and potentially 27 lives saved (assuming 7.8% of AMI admissions end in death [20]) among Delaware residents during the 2003–2004 post-ordinance period.

In comparison to previous studies, the 2002 Delaware smoking ordinance effect on the incidence of AMI was significantly smaller (9% in Delaware after population adjustment, versus 11% in Piedmont, 27% in Pueblo, 47% in Helena and 17% in Toronto, respectively) [5–8]. Nevertheless, Delaware may have been experiencing health benefits like decreased rates of AMI and asthma since 1994 due to its partial smoking ban, and in 2002, when the ordinance became comprehensive, the health benefits for the state would be only modestly augmented.

The smaller effect on the size and rate of AMI may also be explained in part by a change in its clinical definition, which took place during our study period. In 2000, the European Society of Cardiology/American College of Cardiology changed the diagnostic criteria for the definition of AMI to include the use of troponins [21,22]. Consequently, a 2006 study applying the new criteria found that the inclusion of troponins in the definition resulted in a large increase in the annual number of patients diagnosed with AMI [23]. Although this new definition was released in 2000, its gradual increased use over our study period may have blunted the observed effect of the comprehensive smoking ordinance.

### 4.2. Asthma

There has been little published analysis on the health benefits of non-smoking ordinances on their effects on asthma. Most notably, a recent Canadian Medical Association Journal (CMAJ) article agreed with our study [8]; we found that there was a statistically significant decrease in the incidence of asthma discharges among Delaware residents after the introduction of a statewide non-smoking ordinance. As expected, this effect did not carry over into non-Delaware residents and in fact, our research found the opposite effect. We found that non-Delaware residents were shown to have a large increase in the incidence of asthma discharges during the post-ordinance as compared to the pre-ordinance period. Although there is no clear explanation for this increase, it could be due in part to increases of surrounding populations or other health related factors.

### 4.3. Limitations

Our study did not examine the period before the partial non-smoking ban (*i.e.*, prior to 1994) because such information was not readily available from the Delaware Hospital Discharge Database. Similarly, there was lack of available data for the surrounding states on smoke-free polices and therefore, we were unable to compare our data across neighboring states. Moreover, this study was not able to track the large number of patients with asthma—who may have been treated in the emergency department and then released—that may have been protected from exacerbations by the smoke-free policy. Additionally, there is a limitation for the temporal trend used in this study. Even if national data is available, it may not represent the temporal trend in the State of Delaware as the actual trends may be non-linear. Accordingly, the ordinance effect by may be offset by the linear trend term in the model. We must emphasize that our observational data cannot prove causality, it can only provide statistical association between the variables we analyzed in the present study. Finally, individuals under the age of 35 will likely have a different mechanism for AMI — such as a congenital defect—meaning when these cases are combined with older adults, it may create a heterogeneous group that may respond to SHS differently. It is recommended that future studies can perhaps address and compare all three periods in the life of the Delaware Clean Indoor Air Act or similar acts: with the pre non-smoking ban, partial non-smoking ban, and comprehensive non-smoking ban being analyzed.

## 5. Conclusions

The results of our study can be added to the growing list of scientific findings from other studies which demonstrate the real and practical cardiovascular health benefits gained by initiating comprehensive anti-smoking ordinances. It showed that there is very little question regarding the added health benefits that can be gained by strengthening existing smoking bans. Moreover, we were able to demonstrate an additional advantage offered by comprehensive non-smoking ordinances by effectively and meaningfully displaying a statistical association in the reduction of asthma discharge rates. This unique finding, added to the cardiovascular health benefits already recognized and widely reported in the scientific literature, may encourage more communities and regions to join those who currently have comprehensive non-smoking ordinances and potentially experience additional health benefits as shown in this paper.

## Figures and Tables

**Table 1:** Comparison of Delaware to Non-Delaware Resident Quarterly and Annual Incidence of Acute Myocardial Infarction.

Delaware Residents						Non-Delaware Residents				
Year	Quarter 1	Quarter 2	Quarter 3	Quarter 4	Annual Total	Quarter 1	Quarter 2	Quarter 3	Quarter 4	Annual Total
1999	435	430	415	453	1733	83	76	97	67	323
2000	455	421	417	468	1761	66	82	84	72	304
2001	479	495	506	465	1945	65	100	102	103	370
2002	475	430	425	*438-not included*	1768	97	102	104	*98-not included*	401
2003	463	439	440	473	1815	81	87	94	92	354
2004	438	429	384	375	1626	81	94	83	67	325

**Table 2:** Pre-ordinance *vs.* Post-Ordinance Quarterly Rates of AMI and Asthma for Delaware and Non-Delaware Residents.

	Pre-ordinance Quarterly Rate*	Post-ordinance Quarterly Rate*	Pre-ordinance vs. Post-ordinance Relative Risk (RR)
Delaware Residents			
AMI	451	430	0.95 (*P*-value 0.022, 95% CI 0.91–0.99)† 0.91 (*P*-value <0.001, 95% CI 0.87–0.95)‡
Asthma	276	278	0.99 (*P* -value 0.770, 95% CI 0.94 to 1.04† 0.95 (*P-*value 0.038, 95% CI 0.90–0.99)‡
Non-Delaware Residents			
AMI	87	85	0.98 (*P*-value 0.726, 95% CI 0.90–1.08)†
Asthma	28	47	1.62 (*P*-value <0.001, 95% CI 1.41–1.86)†

* Not adjusted for population or seasonal effect;

† Non-population adjusted analysis;

‡ Population adjusted analysis

**Table 3:** Comparison of Delaware to Non-Delaware Resident Quarterly and Annual Incidence of Asthma.

Delaware Residents						Non-Delaware Residents				
Year	Quarter 1	Quarter 2	Quarter 3	Quarter 4	Annual Total	Quarter 1	Quarter 2	Quarter 3	Quarter 4	Annual Total
1999	289	278	289	344	1200	19	19	28	34	100
2000	271	242	202	348	1063	21	23	24	45	113
2001	297	296	218	334	1145	32	27	16	46	121
2002	298	206	230	305-*not included*	1039	31	31	30	*35-not included*	127
2003	244	291	266	375	1176	40	32	45	73	190
2004	253	240	223	336	1052	59	41	37	50	187
